# An Overview of the Challenges and Progress of Synthesis, Characterization and Applications of Plugged SBA-15 Materials for Heterogeneous Catalysis

**DOI:** 10.3390/ma14175082

**Published:** 2021-09-05

**Authors:** Mozaffar Shakeri, ZeynabAlsadat Khatami Shal, Pascal Van Der Voort

**Affiliations:** 1 Laboratory of Heterogeneous Catalysis, Department of Chemical and Petroleum Engineering, Chemistry and Chemical Engineering Research Center of Iran, P.O. Box 14977, Tehran 16363, Iran; zeynabkhatami@gmail.com; 2 Center for Ordered Materials, Organometallics and Catalysis, Ghent University, Krijgslaan 281-S3, 9000 Ghent, Belgium

**Keywords:** SBA-15, plugged mesochannels, characterization, heterogenization, homogeneous catalysts

## Abstract

A new generation of SBA-15, plugged SBA-15, was initially synthesized in 2002 using extra silica precursors (Si/organic template molar ratios ≈ 80–140) in the gel mixture. The plugged SBA-15 materials possess short cylinders (length ≈ 20–100 nm), which are connected to neighbors by constricted entrances (windows) through the central axis. The gas adsorption–desorption isotherms of plugged SBA-15 materials present unique hysteresis loop Type H5 classification identified by IUPAC in 2015, which is related to certain pore structures containing open and plugged mesopores. The plugged SBA-15 has been used to support various types of catalysts, including metal complexes, metal nanocatalysts, and active metals by the incorporation in their framework demonstrating excellent (enantio)selectivity, stability against coke, and thermal stability. The plugged SBA-15 materials bear the other unique properties of the ship-in-the-bottle synthesis of, e.g., metal complexes that confine homogeneous catalysts, which is not possible by conventional SBA-15 due to leaching. In this mini-review, the challenges and progress of the synthesis in controlling the plugging and incorporation of metals and organic moiety in their framework, characterizing the short mesochannel dimensions (window and length sizes) by several advanced techniques and applying plugged SBA-15 materials in heterogeneous catalysis for challenging reactions, has been discussed.

## 1. Introduction

**Figure 1:**
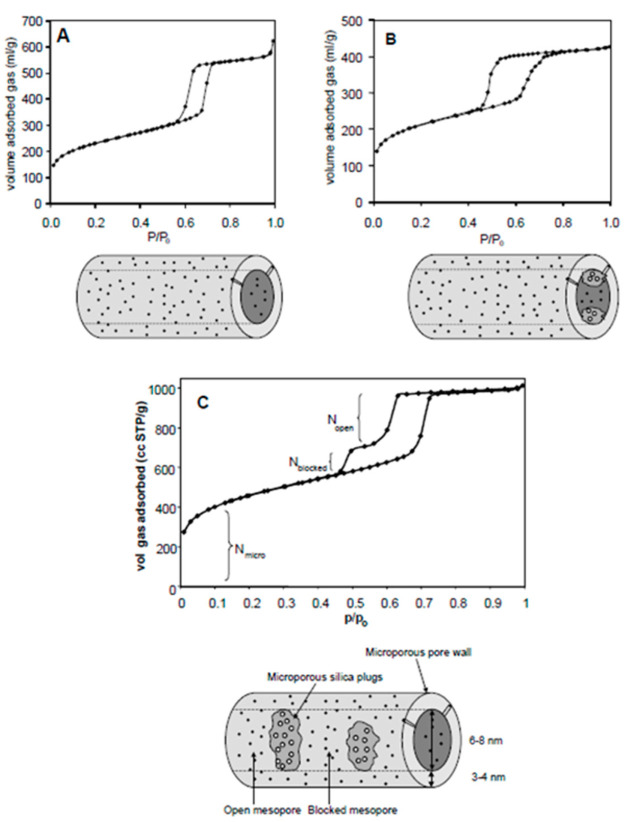
N_2_ adsorption–desorption isotherms (upper) and schematic (lower) of (**A**) conventional SBA-15, (**B**) completely plugged SBA-15, and (**C**) partially plugged SBA-15 materials [11,13].

The silica-based ordered mesoporous Santa Barbara Amorphous (SBA) materials have been widely used as catalysts, catalyst supports, and for the fundamental study of designing efficient catalysts [1]. SBA-15, the main subset of these materials, are microsized particles containing parallel open mesochannels with tunable and narrow pore size distribution in a wide range of 6–12 nm, large surface area and pore volume, wall thickness of about 3–4 nm, and higher mechanical stability than the analogous MCM-41 [2]. Among silica-based materials, SBA-15 have found the most applications in catalysis for production of fine and commodity chemicals, [3,4] adsorption, [5,6] separation [7], energy storage [8], and drug delivery [9]. Large-scale applications of these materials in catalysis, however, are rare mainly due to their limited hydrothermal stability [10]. More specifically, materials that were prepared below 100 °C already showed a decrease in their microporosity upon treatment in water even at room temperature [2]. Furthermore, the conventional SBA-15 has no capability to retain confined homogeneous catalysts, e.g., metal complexes that are very important in synthesis of fine chemicals and enantiopure organic compounds. The search for more stable SBA-15 with increased microporosity resulted in a new generation of SBA-15 with improved structural properties, which was named plugged hexagonal templated silicas (PHTS, hereafter referred to as “plugged SBA-15”) [11,12]. The plugged SBA-15 contains short mesochannels with windows smaller than the mesopore sizes (Figure 1B,C), and they are aligned through the central axis [11]. These properties of plugged SBA-15 have resulted in superior catalytic performances and unique capabilities, such as the ship-in-the-bottle synthesis of homogeneous catalysts, [12] which was not possible by open mesochannels of SBA-15 (Figure 1A) [13]. Since the first synthesis of plugged SBA-15 in 2002, many efforts have been made to improve the synthesis methodology, incorporation of active metals catalysts and (functional)organic moieties in their framework, characterization of the physicochemical properties, and exploration of their applications. No review addressing these materials has yet been reported. The challenges and progress of synthesis, characterization, and applications of plugged SBA-15 in catalysis in comparison with conventional SBA-15 materials are reviewed.

## 2. Challenges and Progress of Synthesis of Plugged SBA-15

### 2.1. Background and Mechanism of (Plugged) SBA-15 Synthesis

The SBA-15 materials are synthesized in the presence of a copolymer of poly(ethylene oxide)-b-poly(propylene oxide)-b-poly(ethylene oxide) (PEO-PPO-PEO) triblock copolymers (Pluronic P123) and a silica source in a highly acidic aqueous solution through the cooperative self-assembly (CSA) mechanism to form an inorganic-organic mesostructured composite [14]. This mechanism includes the three steps of the formation of the spherical micelles of a templating agent followed by inorganic–organic composite formation, their transformation into cylindrical micelles, and finally, aggregation of these micelles into two-dimensional hexagonal structure followed by precipitation [14]. Based on the CSA mechanism, the majority of PEO chains of the organic templates insert into the silica frameworks, generating microporosity after their removal by calcination [15]. In 2002, Van Der Voort et al. introduced a new generation of SBA-15 for the first time, plugged SBA-15, which possesses silica plugs inside the mesochannels (plugged mesochannels; Figure 1B,C) [11,13].These mesochannels are aligned through the central axis, possessing bigger wall thickness and enhanced microporosity [11]. The plugged SBA-15, similarly to conventional SBA-15, is synthesized by dissolving organic template of Pluronic P123 in an aqueous acidic solution, followed by the addition of increased amount of silica sources (TEOS/P123 ≈ 84–146) and aging at various temperatures. The N_2_ adsorption isotherms of plugged SBA-15 materials are similar to those of conventional SBA-15 [11]. The desorption isotherms, however, are different and present either one- or two-step delayed desorption upon the plugging of mesochannels (Figure 1B,C) [11]. The plugged SBA-15 materials possess less pore volume, less surface area, and smaller pore size but enhanced microporosity and bigger wall thickness in comparison with SBA-15 synthesized under similar conditions [15]. A few groups then investigated the origin of plugging of SBA-15 materials to control its formation. Van Der Voort et al. concluded that the plugs are made by templating by low molecular weight impurities such as single or diblock copolymers or even free PO chains in the Pluronic P123 [13]. However, the study by Kruk et al. suggested that, when the TEOS/ethylene oxide (EO_n_) molar ratios are high, only a fraction of available TEOS interacts with the EO_n_ blocks to form the SBA-15 structure, and the rest of TEOS hydrolyzes and condenses inside the mesochannels forming the plugs [16]. Based on this mechanism, metal–organic chemical vapor deposition (MOCVD) was used to reduce the pore mouth of the pristine SBA-15, and the obtained materials were characterized by N_2_ sorption, TEM, and XRD [17]. It was concluded that the materials obtained by the MOCVD technique showed plugging of the mesochannels with restricted entrance sizes while keeping higher textural properties (pore volume, surface area, and pore size), which possibly results from the deposition of the metal–organics mainly on the pore mouth rather than in the internal sections [17]. The aforementioned studies thus all agree that plug formation is controlled by the CSA mechanism.

### 2.2. Progress of the Synthesis of Silica-Based Plugged SBA-15

Van Der Voort et al. investigated control over the percentage of the mesochannels plugging (V_mesopore-plugged_/V_total mesopore_ × 100) from 0% to 100% upon an increase in the TEOS/Pluronic P123 molar ratios in the range of 59–146 [11]. Following this study, several research groups have endeavored to improve the procedures of synthesis, aiming for cost-effective synthesis with a facile control of plugging, particles morphology and pore geometry, and active incorporation of metal atoms and organic moieties in the silica framework (Table 1). For example, the use of different silica precursors has been investigated, as they affect the plug formation and the economic viability of large-scale production. The synthesis of plugged SBA-15 through a two-step process using the inexpensive silica precursor of sodium metasilicate pentahydrate in the presence of NaCl resulted in partially plugged SBA-15 materials with a percentage of plugging ≈ 15% and microporosity of 25% of the total volume [18]. In another study, Wang et al. demonstrated the preparation of plugged SBA-15 with plugging the mesochannels about 10% with cheap sodium silicate (Si/Pluronic P123 ≈ 50) by dividing the silica precursor addition to the synthesis mixture [19]. Interestingly, the synthesis of partially plugged SBA-15 materials was also achieved through the one-step addition of sodium metasilicate nonahydrate (Si/Pluronic P123 ≈ 60), and using various alcohol amines as the aggregation agent of the silica source caused plugging of the mesochannels to about 10% (Table 1) [11,20]. The plugging inside the mesochannels of SBA-15 and the amount of microporosity were tailored by employing various alcoholamines and aging temperatures [20]. De Jong’s group reported a new synthesis procedure using a one-time slow addition of TEOS (Si/P123 ≈ 120) to the synthesis mixture over a wider range of conditions (temperature and aging period) for the tailoring and assessment of the window sizes of plugged SBA-15 materials [21]. This new procedure of the synthesis resulted in “completely” plugged mesochannels of 100% (Table 1) over a wider range of both windows (≈2–5 nm) and mesochannel sizes (≈5–9 nm) [21]. The level of plugging of SBA-15 materials could be tailored from partially to completely by the amount of polyvinyl alcohol (PVA) by a dual templating strategy employing simultaneous PVA and P123 at the TEOS/P123 molar ratio of 120 [22]. The advantage of using PVA was the acquisition of a larger (external) surface area with a significant amount of microporosity (10% relative to the total pore volume) in a wide range of plugging. These synthesis efforts have led to improved plugged SBA-15 synthesis with controlled properties.

**Figure 2:**
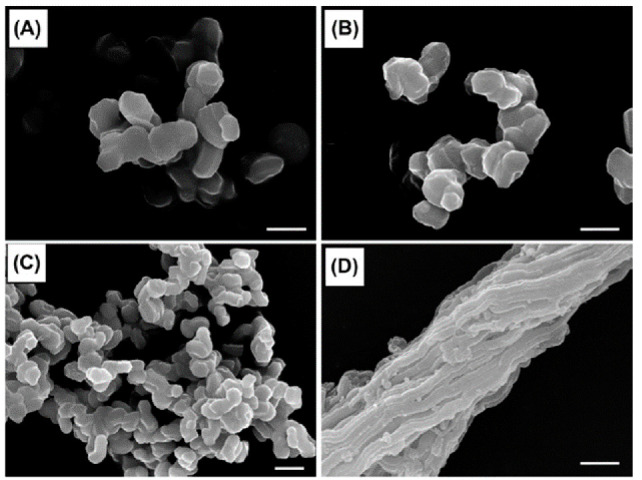
SEM images of various plugged SBA-15 materials of (**A**) 70 °C 1 day, (**B**) 70 °C 3 days, and (**C**) 80 °C 3 days synthesized through a one-step TEOS addition under static condition during the aging period [21], and (**D**) the plugged SBA-15 sample synthesized under stirring conditions during the aging period [26]. The scale bars are 3 µm.

**Figure 3:**
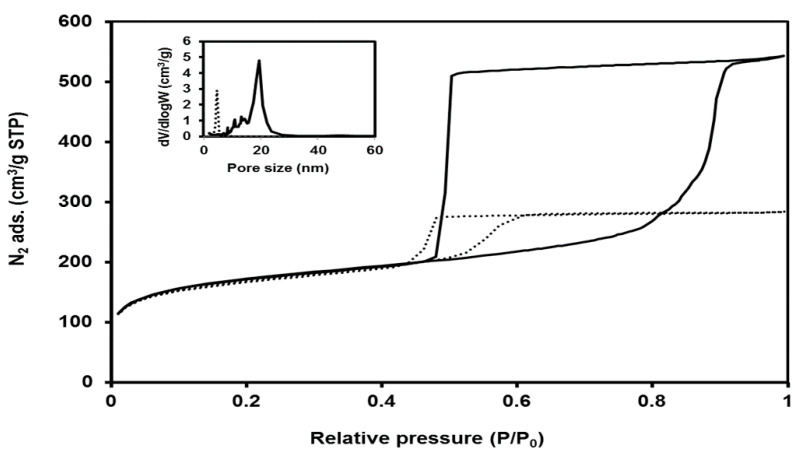
N_2_ adsorption–desorption isotherms of an m-MCF material, its pore size distributions (insert; full line), and the original plugged SBA-15 (dotted line), which were synthesized at 70 °C 3 days [29].

Van Bavel et al. investigated the morphology changes of plugged SBA-15 particles by changing the temperature of the initial stage of the gelation and TEOS/Pluronic P123 molar ratios [26]. The microsized particles morphology evolved from fibrous-like rods at 60 °C to rough rods with deposits of small particles at 70 °C and eventually to spheres at 80 °C at the TEOS/Pluronic P123 molar ratio of 120 [26]. The synthesis of SBA-15 with the partial plugging of mesochannels of less than 10% with platelets morphology was reported by taking advantage of the partitioned cooperative self-assembly (PCSA) mechanism by the TEOS addition at various interval times to the gel mixture (Table 1) [23]. The fine tuning of the amount and time intervals of the two-stage TEOS addition led to control over the transformation of the plugged SBA-15 from the planes to UFO-shaped morphology [23]. The image analysis of plugged SBA-15 by the one-time TEOS addition method followed by aging under static conditions resulted in short rod-like morphology of about 1–3 µm, which is more interesting for catalytic applications in comparison with the fiber-like morphology obtained through conventional synthesis with stirring over the aging period (Figure 2) [21]. The relatively dispersed short rod-like morphology might originate from static conditions during aging that prevented particle attachment [27]. In addition to the morphology of the particles, changes in the size and shape of the mesochannel were investigated [28]. Addition of the swelling agent 1,3,5-trimethylbenzene into the synthesis mixture of plugged SBA-15 led to the transformation of 2D plugged mesochannels to 3D large cavities (12–20 nm), where each cavity is connected to the neighbors by twelve small windows (2–5 nm) [29]. The obtained material was named modified mesocellular foam “m-MCF”. The N_2_ isotherms of the m-MCFs have shown much higher N_2_ adsorption and broader hysteresis loops compared to plugged SBA-15, indicating a much higher porosity (Figure 3). The desorption isotherm of m-MCFs shows capillary evaporation at the relative pressure of about 0.49. This is similar to plugged SBA-15, which is an indication for “ink-bottle”-type pores consisting of large cavities connected by small windows (≈≤5 nm) (Figure 3) [29]. Consequently, tailoring the morphology and cavity dimensions over a wide range of sizes was achieved by tailoring the synthesis conditions and by the use of additives.

### 2.3. Synthesis of Plugged PMO SBA-15 Materials

**Figure 4:**
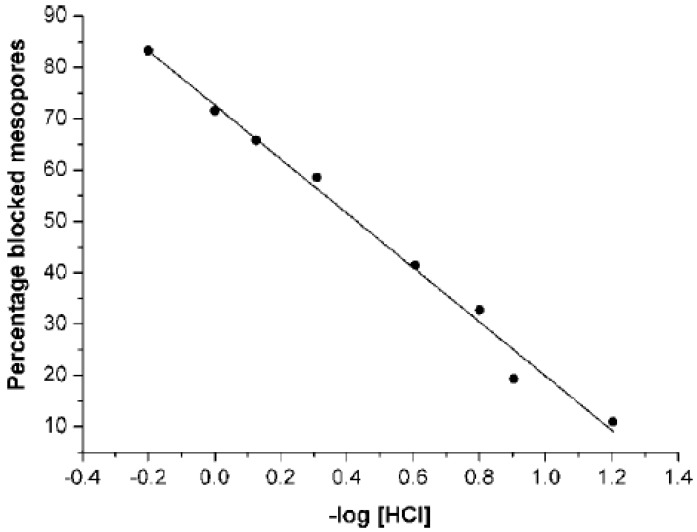
Percentage of blocked mesopores calculated from the nitrogen desorption isotherms using NLDFT as a function of the −log [HCl] [24].

Periodic Mesoporous Organosilicas (PMOs) containing organic moieties in their framework are prepared by condensing a hydrolysable bis-silane around an organic template [30]. The bis-silane “Z_3_Si–R–SiZ_3_” possesses an organic functional linker between the silicon atoms (R = –CH=CH–) and an ethoxy or a methoxy group (Z) connected to the silicone atom. The use of bis-silane of 1,2-bis(triethoxysilyl)ethylene (BTESE) instead of the pure silica precursors in the presence of the Pluronic P123 template in an acidic medium resulted in PMO SBA-15 materials [30] with SBA-15 symmetry in a mechanism similar to the formation of an SBA-15 material [1]. The PMO materials present excellent properties of tunable surface hydrophobicity and enhanced mechanical and hydrothermal stability by the incorporation of organic moieties in their framework [31]. In 2009, Van Der Voort’s group reported the synthesis of both conventional PMO SBA-15 with open mesochannels and partially plugged PMO SBA-15 by organosilicas of the E-isomer of 1,2-bis(triethoxysilyl)ethane and understood that the plugging of PMO SBA-15 materials occurs in pHs < 1 [32]. The Van der Voort group furthermore investigated the systematic plugging of PMO SBA-15 materials with 100% E-configured ethenylene bridges by adjusting the acidity/pH. They achieved plugging of the mesochannels accurately in a wide range of 5.4–83.3% (V_meso-blocked_/V_mesopore_) by change in the pH of synthesis, as shown in Figure 4 [24]. The reasons behind the dependency of plugging on the pH values was raised by the alteration in hydrophobic/hydrophilic volume ratios of the organic templates by the transformation of globular pores at very low pH values into a 2D hexagonally ordered structure at higher pH values [24]. Lin et al. reported the synthesis of the PMO SBA-15 type with ethane-bridged groups by employing H_3_PO_4_ acid to tune the pH in the range of 0.5–2. The results showed that the polyprotic weak acid H_3_PO_4_ is preferable for the synthesis of partially plugged SBA-15-type ethane-bridged PMOs with larger pore sizes and surface areas under mild acidic conditions [33]. Plugged PMO SBA-15 containing an organic moiety of either 1,2-bis(triethoxysilyl)ethane (BTESE) or 1,2-bis(trimethoxysilyl)ethane (BTMSE) were investigated between SiO_2_/P123 molar ratios of 30–290, resulting in enhancement of the microporosity and tailored plugging of the mesochannels from partially to completely plugged [25]. In 2015, Karimi et al. synthesized partially plugged bifunctional PMO SBA-15 and open PMO SBA-15 materials with variable contents of bridged IL-phenyl or -ethyl units at SiO_2_/P123 ≈ 80 in acidic media [34]. The results thus demonstrated the possibility of plugging the mesochannels of PMO SBA-15 by control of the hydrolysis and the pH.

### 2.4. Synthesis of Metal Substituted Plugged SBA-15 Materials

Incorporation of metal heteroatoms like Al into the framework of silica-based mesoporous materials is essential to promote their Brönsted and Lewis acidities [35,36,37]. However, incorporation of Al into the silica wall is challenging, as both species of the silica and aluminum possess a positive charge under the highly mineral acidic conditions of synthesis. Yang et al. reported the synthesis of partially plugged AlSBA-15 through the hydrothermal process using spent fluidized cracking catalyst (sFCC) zeolites [38]. The use of sFCC as the single source of both silicon and aluminum resulted in partially plugged AlSBA-15, with Si/Al of about 31 resulting from the increased pH of the synthesis mixture due to the acidic site of silanol groups enhancing Al incorporation in the framework. A simple and flexible method for the incorporation of Al into the silica wall of SBA-15 materials is using aluminum salts or a highly diluted HCl solution to provide the Al precursor under milder acidity [39,40,41]. Aluminum nitrate provides weak acidity around the isoelectric point of silica (pH ≈ 2), allowing the incorporation of aluminum; however, this is associated with the disadvantage of slow hydrolysis of silica and, thus, low yields of solid materials. The plugged AlSBA-15 with an Al content of 0.7–3.0 wt% was prepared in an aqueous solution within a narrow pH 1.4–2.8 containing triblock copolymer templates, aluminum nitrates, and a silica source without using extra amount of silicon. This resulted in the excellent adsorption of isopropyl alcohol and the catalytic decomposition of nitrosamine [39]. The plugged AlSBA-15 materials obtained under acid-free conditions exhibited stronger acidity than conventional AlSBA-15 materials as a result of aluminum migration and rearrangement to the surface and due to structural reconstruction during the hydrothermal treatment to form plugs, promoting tetrahedral-coordinated Al species with high accessibility [40,42,43]. In addition to Al incorporation, vanadium-containing SBA-15-type ethane-bridged PMOs (1.8 wt%) were successfully prepared through a direct synthesis approach under mild acidic conditions by H_3_PO_4_ [33]. It is worth mentioning that a high Al content in SBA-15 was achieved via a two-step hydrolysis-controlled approach [44]. In this method, TEOS was initially hydrolyzed at lower pHs, followed by the addition of aluminum isopropoxide when the white solid precipitate started. Hereafter, the pH was increased to 7, resulting in materials with a medium Bronsted and Lewis acidity and a Si/Al ≈ 20 [44]. The plugged MSBA-15 (M = Al, V,…) showed stronger acidity compared with the conventional MSBA-15, suggesting the importance of a further exploration of heteroatom substitutions for challenging reactions.

## 3. Stability Study of Plugged SBA-15 Materials

Mechanical and hydrothermal stabilities of porous materials are crucial for their efficient catalytic performances. The plugged SBA-15 materials have shown superior stabilities compared to conventional SBA-15. For example, the plugged SBA-15 retained about 80% of its pore volume when exposed to a pressure of about 800 mega pascals (MPa); however, the conventional SBA-15 retained only about 70% of its pore volume even at the pressure of about 500 MPa [11]. The most challenging aspect of SBA-15 materials is their hydrothermal stability. The hydrothermal stability of plugged SBA-15 in comparison with conventional SBA-15 was systematically investigated by treating these materials in hot water at 100 °C for several days [45]. The gas adsorption–desorption analysis showed a gradual increase in the mesopore sizes by broadening the pore size distribution in all the cases. For example, treatment of the conventional SBA-15 in hot water at 100 °C for 8 days led to an increase in the pore size from 10.3 to 12 nm, a significant decrease in the microporosity, and a 45% decrease in the BET surface area [45]. In comparison, for the plugged SBA-15 samples prepared according to a standard procedure using a TEOS/P123 molar ratio of 120, the plugs persisted in the hydrothermal treatment. Nevertheless, an increase in the pore size from 8.9 to 10.9 nm and a 38% decrease in the BET surface area was noted [45]. The samples prepared at lower temperature and a shorter time of aging; the plugs were nearly completely eliminated after 4 days of boiling, indicating that the stability of the plugs depends on the synthesis conditions [45]. These results have shown that hydrothermal stability is still a major challenge of (plugged) SBA-15 materials. The synthesis of (plugged)SBA-15 at higher temperatures to eliminate microporosity [2], calcination at higher temperatures to reduce silanol groups, and surface functionalization with alkyl groups might be promising approaches to enhance the (hydro)thermal stability.

## 4. Characterization of Plugged SBA-15 Materials

**Figure 5:**
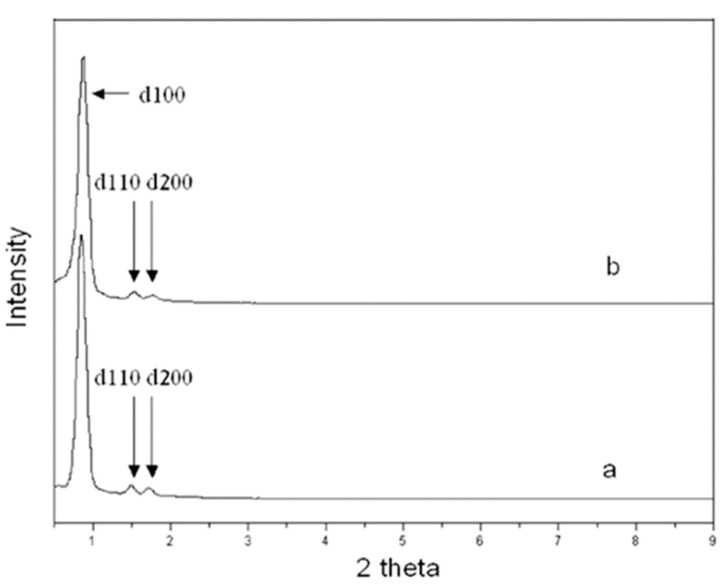
Small angle X-ray scattering patterns for calcined (**a**) SBA-15 and (**b**) plugged SBA-15 [18].

**Figure 6:**
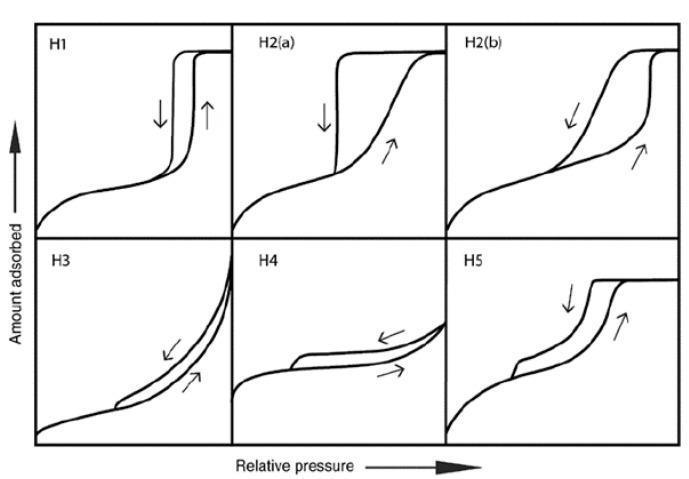
Updated classification of hysteresis loops by IUPAC 2015 [48]. The Type H1 loop indicated for uniform mesopores materiarls (e.g., MCM-41, MCM-48, SBA-15), the Type H2(a) loop indicated for pore-blocking with narrow neck sizes (e.g., vycor., SBA-16 and KIT-5 silicas), the Type H2(b) loop indicated for pore-blocking with larger neck sizes (e.g., MCF), the Type H3 loop indicated for non-rigid aggregates of plate-like particles (e.g., certain clays), and the Type H4 loop indicated for aggregated crystals of zeolites and micro-mesoporous carbons. The Type H5 hysteresis loop indicated for plugged SBA-15 materials.

The major characterization challenges of plugged SBA-15 are, firstly, differentiating them from conventional SBA-15 and, secondly, sizing the window (entrance) and the length of the plugged mesochannels (Figure 1B,C). The plugged SBA-15, similarly to the conventional SBA-15 materials, presents three diffraction peaks at (100), (110), and (200) planes at a low 2θ angle, showing a typical p6mm XRD pattern (Figure 5) [18]. The peak intensity of the plugged SBA-15 is weaker than those of conventional SBA-15 due the presence of plugs inside the pores, which decrease the difference in electron density between pores and silica wall. However, this is not enough to differentiate between plugged and conventional SBA-15 materials [46]. The gas adsorption–desorption of, e.g., N_2_ isotherms is a strong technique for determining the possibility of the plug formation in mesochannels of SBA-15. The N_2_ adsorption isotherms of the plugged SBA-15 and conventional SBA-15 materials show step capillary condensation at the relative pressures of about 0.6–0.9, suggesting ordered mesochannel sizes of larger than 5 nm (Figure 1). The shape of the hysteresis loop isotherms of plugged SBA-15, however, is significantly different than that of conventional SBA-15 (Figure 1). The desorption isotherms of plugged SBA-15 present either one- or two-step capillary evaporation closing at the lower relative pressures (Figure 1B,C). If one-step N_2_ capillary evaporation occurs at the relative pressure (*P/P*_0_) of about 0.4, this is called “completely plugged SBA-15” (Figure 1B), suggesting that all mesochannels contain plugs. However, if two-step N_2_ desorption is observed, this is called “partially plugged SBA-15”, meaning that only a fraction of the mesochannels contain is plugged and the rest of the mesochannels are open (Figure 1C) [13]. The desorption isotherm of plugged mesochannels is delayed until the vapor pressure is reduced below the stability limit of liquid nitrogen. This occurs at *P/P*_0_ of about 0.4, where evaporation does not depend on the pore size and pore geometry, and thus, window size measurements from the desorption isotherm are not possible [47]. Indeed, the hysteresis loop of plugged SBA-15 is different from than that of the other porous materials, and this led to receiving its own hysteresis loop classification of Type H5 by IUPAC in 2015 (Figure 6, H5) [48]. This is because the hysteresis Type H5 loop is related to certain pore structures containing both open and partially blocked mesopores, which is different from the Type H1 loop for conventional SBA-15 materials with a narrow range of mesoporous materials. Ryoo et al. investigated the possibilities of plug sizes and micropore interconnectivity of the mesochannels when combining inverse carbon replication and gas physisorption techniques. The gas physisorption showed that a sample that was completely plugged according to the nitrogen adsorption was only partially plugged according to the argon adsorption [16]. The results of gas physisorption by different gases thus provided information about the possibility of plug formation and percentage of mesochannel plugging [16]. The micropore volume is the other important factor of (plugged) SBA-15, which has been characterized by the use of the *t*-plot method from gas sorption isotherms [49]. The characterization results demonstrated that a gas adsorption–desorption analysis is a strong technique to differentiate plugged SBA-15 from conventional SBA-15 and to determine the physical properties. However, this technique is unable to directly size the length and the window of the plugged mesochannels.

**Figure 7:**
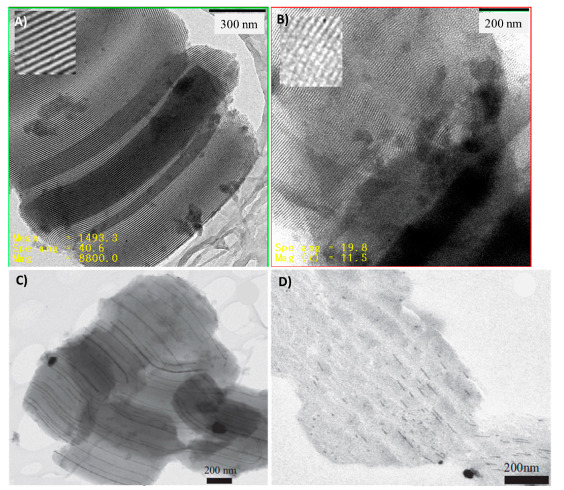
Bright field TEM images of (**A**) open SBA-15 and (**B**) plugged SBA-15 [13] and HRTEM images of silver nanowire in (**C**) conventional SBA-15 (**D**) plugged SBA-15 [46].

Understanding the window sizes of plugged mesochannels of SBA-15 is extremely important for their various applications [21]. The N_2_ sorption isotherms, transmission electron microscopy (TEM), atomic layer deposition (ALD), and surface modification by alkoxysilanes are the most used techniques to estimate the window sizes of ‘‘ink-bottle’’ materials. The sizing of plugs (plug size = mesopore size—window size) via TEM is very challenging, because there is almost no difference between the diffraction contrast of plugs and that of the walls of mesochannels. The use of ALD technique requires extensive cycles of oxide deposition, making this technique practically very difficult [50]. This is because, in the ALD technique, in each cycle, a layer of the metal oxides such as TiO_2_ and HfO_2_ are deposited on the porous materials, and then, the obtained materials are characterized by complementary techniques such as porosimetry, X-ray reflectivity, X-ray fluorescence, and TEM [50]. Once the characterization showed complete plugging of the ink-bottle mesoporous materials, the window size (neck) was estimated by the number of ALD cycles and thickness of the atomic layer. The surface modification by alkoxysilanes followed by an analysis by the gas sorption technique was found to be a promising approach to determining the window sizes of SBA-16 materials with a cage-like structure [51]. Therefore, the systematic study of sizing of the windows of plugged mesochannels of SBA-15 by surface modification with alkoxysilanes, the silylation technique, was conducted by the De Jong’s group [21]. The plugged SBA-15 materials obtained in various synthesis conditions were silylated by alkoxysilanes with different alkyl chain lengths, and these were exposed to N_2_ sorption. The window size of plugged SBA-15 material was calculated from the shortest alkyl chain length of the alkoxysilane-blocking N_2_ adsorption by the formula of “window size = 0.75 + 2 × (n − 1) × 0.125 [nm]”, where n is the number of carbon atoms of the alkyl group [21,52]. The window sizes of plugged SBA-15 materials were approximately 2.3, 2.6, and 3.0 nm for the materials synthesized at 70, 80, and 90 °C for 1 day, respectively, compared to 3.1 and 3.9 nm for plugged SBA-15 synthesized at 70 and 80 °C for 3 days [21]. The mesochannel dimensions of conventional SBA-15 and plugged SBA-15 materials were investigated by TEM in bright field transmission mode using microscopy. Obtained images of the side view of mesopores in SBA-15 show running smoothly over several micrometers of length, while the PHTS displays smaller domain sizes for the ordered mesopores (Figure 7A,B) [13]. De Jong’s group further investigated sizing of the length of the plugged mesochannels by the synthesis of silver nanostructure by the two-solvent technique and by the deposition of Pt single atoms on the amine-functionalized mesochannels of conventional and plugged SBA-15 materials [12,46]. HRTEM imaging of the silver nanostructure of the sliced plugged SBA-15 materials along the central axis by microtome confirmed a short nanowire with a length of about 20–70 nm. However, the images of the silver nanostructure in conventional SBA-15 material showed long micrometer-sized nanowires through the whole channels, in accordance with the previous results (Figure 7C,D) [46]. The HRTEM investigation of plugged PMO SBA-15 and conventional PMO SBA-15 materials showed short mesochannels and open long mesochannels, respectively [53]. These characterization efforts showed the progress of window and mesochannel sizing by (in)direct approaches.

**Figure 8:**
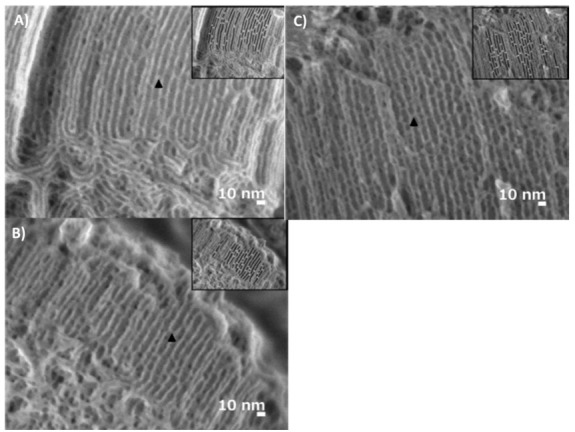
LV-HRSEM images of plugged SBA-15 obtained under different conditions of (**A**) NaCl-0.5 M, (**B**) SBA-15-ref, and (**C**) EtOH-0.5 M. The black arrows show examples of plugs. The inset shows the same image after the analysis, with the pores marked in black and plugs in white. Images were taken with a landing energy of 300 eV after applying a specimen bias of 5 kV [54].

The plugs of SBA-15 materials have been investigated by the low-voltage high resolution scanning electron microscopy (LV-HRSEM) technique [54], presenting cylindrical segments of various lengths separated by plugs and aligned through the central axis (Figure 8) [46,54,55]. The LV-HRSEM investigation of the plugs clearly resolved that they are present in the pores on the external surface of the particles and in the interior of the mesochannels [54]. Later, another group also investigated the plugged SBA-15 by field emission SEM (FE-SEM), visualizing clear short mesochannels with constricted windows along the central axis [23]. These results suggest that the progress of the analytical techniques have now provided the opportunity for the direct observation of plugs.

## 5. Catalytic Applications of Plugged SBA-15 Materials

**Figure 9:**
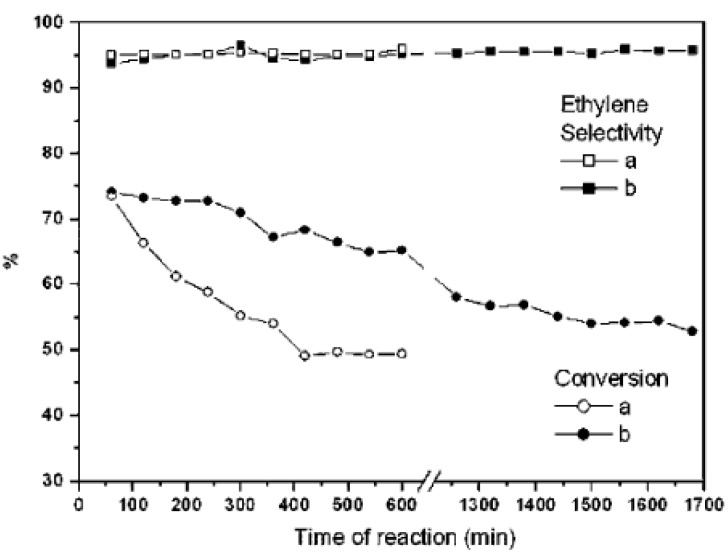
Conversion and selectivity for the hydrodechlorination of 1,1,2-trichloroethane over (**a**) Pd/SBA-15 and (**b**) Pd/plugged SBA-15 [18].

The performances of plugged SBA-15 as the catalyst, catalyst support, and adsorbent have been investigated. The plugged and conventional SBA-15 materials were used for the adsorption of different alkanes [56]. The adsorption study of various alkanes on plugged SBA-15 and conventional SBA-15 materials showed that plugs influenced the uptake of adsorbate at the low relative pressure region as a result of the kinetic diameter and shape of the molecule [56]. The TiO_x_ and VO_x_ synthesis in the mesochannels of plugged and conventional SBA-15 materials were reported by the adsorption of metal complexes of TiO(acac)_2_ and VO(acac)_2_ followed by thermolysis [57]. The amount of both TiO_x_ and VO_x_ species on plugged SBA-15 was lower than that on conventional SBA-15, the difference resulting from a limited diffusion of corresponding metal complexes into constricted windows of the plugged mesochannels [57]. The plugged SBA-15 materials were mainly used for catalytic applications. Lee et al. prepared nanopalladium catalysts on plugged SBA-15 and conventional SBA-15, and they used these for the hydrodechlorination of 1,1,2-trichloroethane into ethylene at 473 K [18]. The PdO/plugged SBA-15 showed a much higher resistance to deactivation in comparison with that on conventional SBA-15, showing only a 20% decrease of its initial activity in 30 h while the PdO/SBA-15 lost 25% of its initial activity in 6 h (Figure 9) [18]. The sustained activity by PdO/plugged SBA-15 originated from smaller sizes of PdO nanoparticles penetrating inside the increased micropososity of the plugged SBA-15 [18], which prevented sintering.

**Figure 10:**
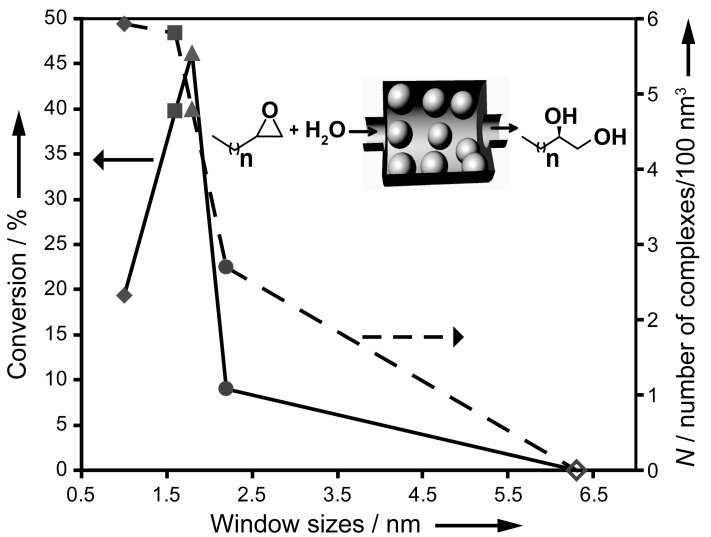
Percent conversion of 1,2-epoxyhexane and N (number of chiral (salen)Co^III^ complexes per 100 nm^3^) versus window size by catalyst 1 (♦), 2 (■), 3 (▲), 4 (●), or 5 (◊). Reaction conditions: catalyst loading of 0.015 mol% (percent molar ratio of confined chiral (salen)Co^III^ relative to 1,2-epoxyhexane), 0.75 equiv H_2_O (relative to 1,2-epoxyhexane), room temperature, and reaction time of 48 h. The inserted scheme shows the HKR of terminal epoxides using a confined chiral (salen)Co^III^ (spheres) inside the plugged nanochannel of SBA-15. The maximum theoretical conversion is 50% [12].

In another study, Oliveira et al. investigated Pd nanoparticles on thiol-functionalized plugged SBA-15, comparing them with aerosol-380, m-MCF, and SBA-16 for liquid-phase Heck and Suzuki reactions [58]. The Pd nanocatalysts/plugged SBA-15 in the cross-coupling Suzuki reaction, which requires stronger alkaline conditions, showed a higher stability than those on SBA-15 and m-MCF as a result of the increased structural stability. A novel heterogeneous catalyst based on the conventional and plugged PMO SBA-15 with imidazolium-based bifunctional organic moiety were used for the oxidation of various benzyl alcohols by Karimi et al. [53]. These catalysts, palladium nanoparticles supported into the nanospaces of imidazolium-based bifunctional plugged PMO SBA-15 and conventional PMO SBA-15, resulted in a drastic change in selectivity toward either benzaldehyde or benzoic acid upon plugging of the mesochannels [53]. The conventional PMO SBA-15 showed a higher activity for the selective oxidation of benzyl alcohol to benzaldehyde, whereas the plugged PMO SBA-15 exhibited a high yield and selectivity to benzoic acid under the same reaction conditions [53]. In another interesting experiment, Park et al. investigated the catalytic performance of L-proline immobilized on plugged SBA-15 compared with the homogeneous one in the important reaction of diethyl malonate addition to cyclohexanone [59]. A significant increase in the activity (32%) and enantioselectivity (72%) was observed by the former. The plugged SBA-15 materials were also used for confinement of various metal complexes by the ship-in-the-bottle synthesis. The systematic study of the confinement of metal complexes of M-salen (M = Co and Fe) by the ship-in-the-bottle synthesis on various plugged SBA-15 with different window sizes in comparison with the other mesoporous materials was intensively investigated for various reactions [12,60]. The conventional SBA-15 did not retain any metal complexes, due to washing out during the ship-in-the-bottle preparation. The plugged SBA-15 showed no leaching of the Co-salen complex, and it exhibited a much higher catalytic performance in the hydrolytic kinetic resolution of various aliphatic terminal epoxides in comparison to the homogeneous counterpart in terms of activity and enantioselectivity. The turnover frequency (TOF) of catalyst 3 (Figure 10) in this study was 35-fold higher than the homogeneous catalyst while presenting an enantioselectivity of >99% towards 1,2-hexandiol [12]. The amount of confined metal complexes and catalytic activity of the plugged mesochannels presented a strong dependency on the window sizes (Figure 10) [12]. Furthermore, sizing the windows facilitated the reproducibility of the confinement of the metal complexes in plugged mesochannels. De Jong’s group further investigated using m-MCF with large cages (12–20 nm) and constricted the window sizes (1–5 nm) for the ship-in-the-bottle synthesis of the Co-salen metal complexes [29]. Similar to that of plugged SBA-15, the loading of Co-salen in various m-MCFs showed strong dependency on the window sizes and reached a maximum of about 73-mg Co-salen/g-support at window sizes of 1.0−1.3 nm [29]. The Co-salen/m-MCF showed substantially higher activity and thermal stability than both the homogeneous one and plugged SBA-15, achieving a turnover number of about 100,000. Co-salen/m-MCF showed an excellent enantioselectivity (99%) for 1,2-hexanediol at a temperature of 90 °C, while the homogeneous counterpart showed only an enantioselectivity of 88% at 60 °C [29]. The plugged SBA-15 has also been used as catalyst by heteroatom incorporation in the pore wall. Reddy et al. incorporated Al in the framework of both plugged SBA-15 and conventional SBA-15 by using AlCl3, and they were used for the isopropylation of *m*-cresol [61]. The catalytic conversions of *m*-cresol were 78.4% and 59.2% by plugged AlSBA-15 and conventional AlSBA-15, respectively, and the higher activity by the former originated from the higher number of medium strength acid sites by the plugging of silica [61]. Recently, we reported the synthesis of plugged and conventional AlSBA-15 materials under acid-free conditions and employed these in the Friedel-crafts acylation reaction of anisole with acetic anhydride [62]. The (plugged) AlSBA-15 was used for the acylation of anisole by acetic anhydride into 4-acetyl anisole, an important ketoaromatic compound in the laundry industry [62]. The plugged AlSBA-15 showed a lower but a more sustained activity in the acylation reaction, which resulted in a doubled productivity in comparison to that of the conventional AlSBA-15. This higher productivity was the result of less coke deposition on the active sites in the plugged AlSBA-15 [62]. In conclusion, the catalytic applications studies demonstrated superior performances of plugged SBA-15 over conventional SBA-15 in terms of activity, (enantio)selectivity, and stability by benefiting from a confinement effect, stronger acidic sites, and controlled number and sizes of the windows.

## 6. Conclusions and Future Perspectives

The synthesis, characterization, and applications of plugged SBA-15 materials have been reviewed since their discovery in 2002. The overview of the literature shows considerable progress in the synthesis and characterization of plugged mesochannels dimensions by the advanced techniques. The progress of synthesis provided control over morphology, particle size, and tailoring the mesochannels dimensions. Advanced characterization via, e.g., HRTEM and HRSEM, made it possible to measure the mesochannel dimensions (window and length). These catalysts showed excellent catalytic performances by benefiting from controlled physical properties and sizing their dimensions. The plugged AlSBA-15 showed stronger acidity compared to the conventional AlSBA-15, suggesting the attractiveness of the exploration of further heteroatom-substituted plugged SBA-15 materials for efficient catalytic applications. The supported catalysts in plugged SBA-15 showed higher stability than those in conventional SBA-15. However, the limited thermal/hydrothermal stability, lower mechanical stability, and weaker acidity of these materials in comparison to zeolites set still hurdles for their application in challenging reactions, such as biomass conversion. In addition, the use of expensive structure directing agents in the synthesis has made the preparation being less economically viable. Therefore, the development of synthesis and post-synthesis procedures and incorporation of tri-and tetra-valent heteroatoms (B, Ga, Fe, Ti, Sn, V, etc.) in the silica wall of plugged SBA-15 are essential to overcome their weaknesses. In addition to that, the degradation of mesoporous materials by abrasion in slurry liquid phase reactions is another challenge for their applications. Work should be carried out on the shaping of these materials to enhance their mechanical and thermal/hydrothermal stability.

## Figures and Tables

**Table 1:** Summary of various strategies to control the plugging of SBA-15 materials.

No.	Si/P123 ^a^	S_BET_ (m^2^/g) ^b^	V_total_ (cm^3^/g) ^c^	V_micropore_ (cm^3^/g) ^d^	Strategy ^e^	Maximum V_meso, plugged_ (%) ^f^	Ref.
1	146	580	0.44	0.17	Si/P123 control	≈100	[11]
2	124 ^c^	640	0.7	-	Two-step silica addition	≈15	[18]
3	116	897	0.73	0.14	One-step TEOS addition	≈100	[21]
4	119	655	0.659	0.061	Dual templating strategy	≈100	[22]
5	50	836	0.85	0.18	PCSA	≈<10	[19]
6	55	783	0.91	0.126	PCSA	≈<10	[20]
7	60	806	1.07	-	Two-step silica addition	≈<10	[23]
8	60	-	-	0.15	pH control	≈83.3	[24]
9	290	961	0.76	0.26	Si/P123 control	≈100	[25]

^a^ The Si/Pluronic P123 molar ratios were used in the synthesis. ^b^ Total surface area obtained by the BET method. ^c^ Total pore volume. ^d^ Micropore volume. ^e^ Strategy used to control the percentage of mesochannels plugging. ^f^ Maximum percentage of mesochannels plugging reported in the related study. -: not reported.

## Data Availability

Data sharing not applicable.
